# SC-framework: A robust and FAIR semi-interactive environment for single-cell resolution datasets

**DOI:** 10.1016/j.isci.2026.116631

**Published:** 2026-06-29

**Authors:** Hendrik Schultheis, Jan Detleffsen, René Wiegandt, Mette Bentsen, Yousef Alayoubi, Guilherme Valente, Micha Frederick Keßler, Brenton Bruns, Dlnija Mirza, Angeline Usanayo, Kristina Mueller, Jasmin Walter, Philipp Goymann, Moritz Hobein, Carsten Kuenne, Mario Looso

**Affiliations:** 1 Bioinformatics Core Unit (BCU), Max Planck Institute for Heart and Lung Research, Bad Nauheim 61231, Germany; 2 Cardio-Pulmonary Institute (CPI), Bad Nauheim 61231, Germany

**Keywords:** single cell, framework, FAIR, python, multiomics, workflow

## Abstract

Single-cell (SC) sequencing technologies have advanced our ability to resolve cellular heterogeneity, yet the analysis of the resulting data remains complex, insufficiently standardized, and difficult to reproduce. Here, we present the SC-Framework, an FAIR-compliant, layered, semi-interactive analysis environment that combines a Python package with a structured series of Jupyter Notebooks to provide a complete, guided, reproducible, and flexible SC analysis workflow, across multiple modalities. The framework ensures traceability and findability via self-documenting data objects and configuration-defined directory structures. Containerized releases support long-term reproducibility, on both local machines and high-performance clusters. Exemplary single-cell RNA sequencing (scRNA-seq) and single-nucleus assay for transposase-accessible chromatin with sequencing (snATAC-seq) analysis retrace published results within a standardized workflow, and benchmarking demonstrates scalability to nearly 1,000,000 cells. The SC-Framework addresses a gap between rigid automated pipelines and flexible but unstructured toolkit-based approaches, by balancing automation with interactivity, making robust SC analysis accessible to a broad range of users.

## Highlights

• FAIR, containerized environment for reproducible single-cell analysis
• Interactive, scaffolded workflows for exploration and interpretation
• Flexible, extensible support across RNA, ATAC, and other modalities
• In-workflow guidance, documentation, and tutorials for a broad user base

## Graphical abstract

**Figure:**
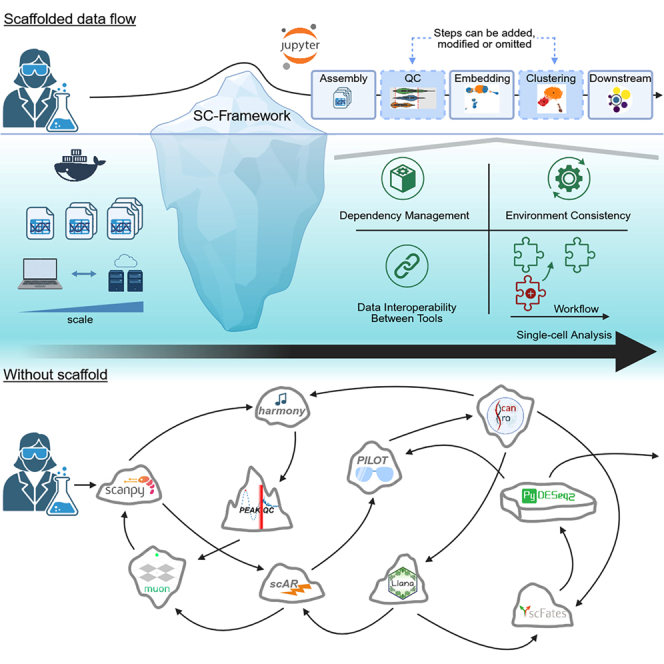

## Teaser

Bioinformatics; Computational bioinformatics; Omics; Transcriptomics

## Introduction

The ever-increasing resolution of sequencing-based methods for various cellular modalities (gene expression, chromatin accessibility, surface protein abundance, etc.) has expanded our understanding of molecular processes, enabling new inquiries into cellular heterogeneity and differentiation at the single-cell (SC) level. However, the growing volume and complexity of SC data present considerable challenges for analysis, requiring sophisticated computational tools and unifying frameworks for reproducible data processing. Notably, methods established for bulk sequencing are not uniformly applicable to SC-resolution data.^1^ While ongoing efforts have led to the development of new tools^2^ and an increasing number of analysis guidelines and best practices workflows,^3^^,^^4^^,^^5^^,^^6^ a fully automated SC analysis workflow akin to those used for bulk data has yet to be achieved.

Between sequencing and final data interpretation, we have identified two major steps: preprocessing and data analysis. Preprocessing encompasses all steps from the initial sequencing reads, basic quality control (QC), cell event identification, as well as mapping and quantification of the reads. The product (in the case of single-cell RNA [scRNA]) is a matrix defined by read counts, with the dimensions corresponding to cells and genes. While the creation of this matrix is sufficiently automated,^7^^,^^8^ the subsequent analysis of this matrix, interpreting its contents, remains challenging and thus the focus of this work.

As of writing, multiple tools aim to provide an environment to simplify and streamline SC analysis (Table S1). Broadly, these tools can be split into four categories: utilities, toolkits, automated workflows, and graphical user interface (GUI) workflows.

Each of these has been designed to appeal to a different audience. Utilities address specific SC tasks, e.g., batch correction (harmony^9^ and ComBat^10^), clustering (leiden^11^ and hotspot^12^), multiplet detection (scrublet^13^ and amulet^14^), trajectory prediction (scFates^15^ and palantir^16^), or annotation (SCSA^17^). Toolkits provide individual analysis steps, offering versatility and flexibility, but often provide minimal guidance for designing a complete SC analysis (Seurat,^18^ Scanpy,^19^ EpiScanpy,^20^ Muon,^21^ squidpy,^22^ SnapATAC2,^23^ scvi-tools^24^). These toolkits require at least minimal coding experience, often rendering them inaccessible for people with a limited computational background. This entry barrier is further increased by the lack of standardization in methods and thresholds across the SC field, which can lead to inconsistent results and hinder reproducibility.^25^^,^^26^ Toolkits are typically aimed at higher-level tasks and often integrate or combine multiple utilities into a working environment, necessitating careful management of computational dependencies. This can complicate long-term maintenance and portability between systems. Automated workflows (e.g., ENCODE workflows for assay for transposase-accessible chromatin [ATAC],^27^^,^^28^ RNA,^28^ chromatin immunoprecipitation [ChIP],^28^ DNA,^28^ and micro RNA [miRNA]^28^ or nf-core pipelines^29^ for scnanoseq^30^ and scrnaseq^31^) require less individual programming but are static, always performing the same steps in the same order. While this benefits reproducibility, it can be limiting for data types and analysis steps that rely on dynamic threshold adjustments, as it is common in most SC workflows. GUI workflows (e.g., WASP,^32^ GRACE,^33^ ASAP,^34^ and SCALA^35^), often web- and cloud-based services, combine the benefits of flexible workflows with a graphical interface. This enables users without programming experience and supports rapid explorative analysis, with immediate feedback on, e.g., parameter adjustments or visualization parameters. However, GUI workflows often have limited long-term reproducibility due to a lack of automation, as well as missing access to latest tool developments, as their integration is typically time-consuming. Further, they often have reduced transparency of analysis implementations and difficulties in scaling or reusing workflows.

An SC analysis framework should therefore prioritize reproducibility, extensibility, flexibility, and usability to address current challenges in the field. However, achieving all four simultaneously is non-trivial, as improvements in one aspect can come at the expense of others. For example, highly flexible systems are often more difficult to reproduce consistently. Consequently, a careful balance between these properties is required to provide an optimal analysis environment. We suggest a semi-interactive workflow design that separates functionality into distinct but interconnected layers to address this challenge. The first layer consists of analytical utilities and toolkits that perform the core computational tasks. A second software management layer handles dependencies, environment consistency, and data interoperability between these tools, while also enabling the implementation and integration of new tools and methods where required to fill functional gaps between existing components. A third layer defines and structures the analytical workflow itself, providing an ordered but flexible framework in which individual steps can be added, modified, or omitted as needed. And a fourth layer delivers a standard software environment that is easy to use, for example through a web browser, while remaining fully scalable in the computational backend to accommodate both small-scale analyses and large high-performance computing setups. These layers are supported by an FAIR-compliant data handling (abbreviation for findability, accessibility, interoperability, and reusability^36^) that ensures systematic recording of parameters, processing steps, and data transformations, thereby enabling reproducibility and traceability across the entire analysis process.

The SC-Framework implements this layered concept and is described in the following.

## Results

### A FAIR single-cell framework providing a guided analysis

**Figure 1:**
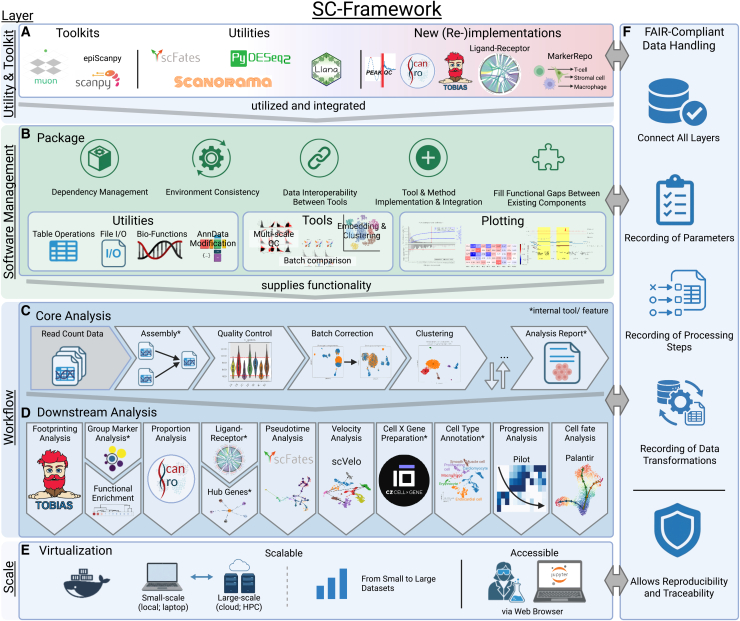
SC-Framework overview The SC-Framework integrates widely used SC toolkits, individual-purpose utilities, and newly implemented SC applications into a unified, layered environment (A). At its core, a Python package provides SC-specific functionality by wrapping and extending the methods in (A), serving as the analytical foundation of the framework (B). This package is leveraged by a structured analysis workflow that guides users through SC data analysis in a reproducible and flexible manner (C and D). The workflow is divided into a core analysis component covering mandatory processing steps (C) and a downstream analysis component comprising optional notebooks tailored to specific biological questions (D). The entire framework is encapsulated within a virtualization environment that standardizes software dependencies and enables deployment across a range of infrastructures, from personal computers to high-performance server clusters (E). Across all layers, automatic logging of data processing steps ensures full traceability and reproducibility of analyses (F). See also Figure S1. Created with BioRender.

The SC-Framework encompasses a collection of utilities and toolkits (including Scanpy,^19^ Muon,^21^ and other scVerse resources; Table S2 and Figures 1A and 1B) packaged into a guided, step-by-step environment providing a comprehensive analysis workflow for RNA, ATAC, and other modalities. Core analysis steps for each modality (QC, batch correction, normalization, and clustering; Figure 1C) are complemented by integrated downstream analysis modules such as annotation, receptor-ligand investigation, and proportion analysis (Figure 1D). A reporting module compiles plots and thresholds into a summary document (Figure 1C; examples in the code repository). Analysis modules are realized as Jupyter Notebooks, combining the reproducibility of automated workflows with the flexibility of toolkits and the explorative capabilities of GUIs. Each notebook follows a predefined structure, with explanatory texts, images, tables, highlighted user input fields, and immutable code blocks (Figure S1). This design allows users to interactively define parameters, store results, and repeat analyses with minimal effort. The automatically generated logs and a hierarchical folder structure further improve reproducibility. The SC-Framework generates plots and corresponding information suitable for inclusion in the methods sections of manuscripts (see code repository).

The SC-Framework adheres to the FAIR software principles,^37^ ensuring that code and data are well-documented, discoverable, openly accessible, interoperable, and reusable (Figure 1). Its design supports reproducibility and interpretability through encapsulation within a versioned, containerized environment (Figures 1B–1F). It is suitable for a broad audience, from novices seeking robust guidance to core facilities managing multiple projects with high reproducibility and throughput requirements.

As introduced above, the SC-Framework implements a layered architecture that separates analysis logic from underlying utilities and hides software dependency management from the user.

The first layer is given by the SC-Framework Python package (Figures 1A and 1B), which provides encapsulated utilities, tools, and plotting functions built on published scVerse software, while extending existing methods and implementing new ones not yet standardized in the field (Figure 1A). Data handling is built around the universal AnnData^38^ format, which stores high-dimensional data and corresponding metadata in a single object. The package is modular, supporting long-term maintenance, adaptation to new data formats, and reuse of routines across related problems such as annotation, embedding, and filtering.

The second layer wraps this package into higher-level analysis modules with interfaces designed for end users, organized as functional pairs of calculation and visualization steps (e.g., a QC module combining cell filtering with global visualization; Figures 1B and S2A–S2C). This layer is deployed through a Conda environment installable with a single command on Linux.

The third layer is given by a series of notebooks split by main topics, ordered according to the analysis flow (Figures 1C and 1D). A command line interface (CLI), included in the layer one package, automates notebook download and sets up the initial folder structure. Each Jupyter Notebook represents one major step of the SC analysis, defined by an input data object, a series of analysis steps, and an export object that is used as input for the successor notebook. Overall, our notebooks follow best practices where possible^3^^,^^5^^,^^6^^,^^39^^,^^40^ and provide alternative branches of commonly used methods when there is no clear consensus. For ease of use, each notebook follows a similar structure with recurring structural elements (Figure S1). At the top, a highlighted cell indicates the need for user interaction and is generally intended for setting analysis parameters such as the organism to be studied, thresholds for analysis, and various parameters that require user input. Most values are preset with defaults (user guidance). Other cells are not intended for editing by default to protect novice users from introducing unintended changes. Marker lists, e.g., cell-cycle stage prediction, sex chromosome genes, rRNAs, and other groups mainly used for various QC steps, are dynamically loaded from within the framework based on the target organism. Each notebook features manual blocks providing information on a specific step, links to additional resources, as well as guidance on the interpretation of the data (e.g., the meaning of QC metrics and what a suitable threshold may look like). Notebooks are accessible locally or via web browser through a Jupyter server that exposes the Conda environment as a kernel, directly linking the managed software environment to the interactive notebook interface.

The SC-Framework includes an introductory video series (https://youtube.com/playlist?list=PLTA07KFyG53ImxJKpoiPO9XiU_I5anMKp&feature=shared) specifically catered to first-time users as an entry to the SC-Framework ecosystem, explaining idioms and structures, as well as guiding users through their first analysis. An extensive manual (https://loosolab.pages.gwdg.de/software/sc_framework/) provides information and examples for advanced users wishing to customize or create new analyses.

The fourth layer applies application virtualization by providing Docker^41^ images for each tested framework release (Figure 1E). These bundle all underlying layers (the Python package, Conda environment, Jupyter notebooks, and server) in a ready-to-use state, deployable as containers locally or on a compute cluster. This ensures that any release can be stably re-executed to reproduce prior data interpretations (information on Docker images can be found in the documentation).

**Figure 2:**
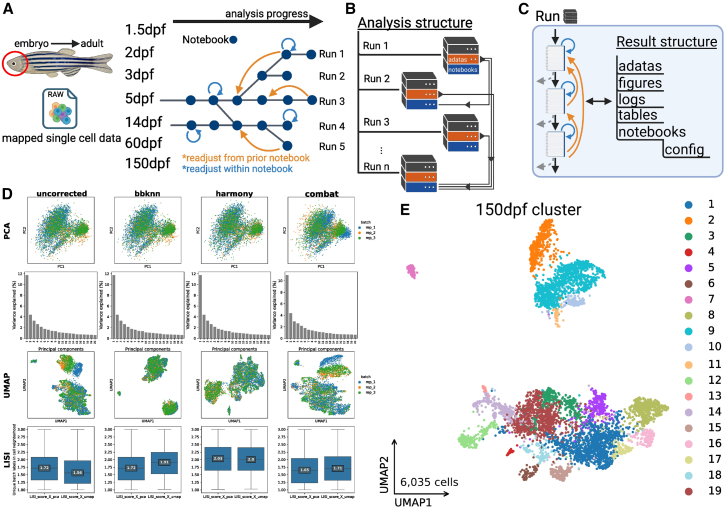
Exemplary scRNA core analysis on zebrafish (A) Zebrafish samples from multiple timepoints are processed by the notebooks. The graph illustrates the progress of an exemplary analysis, showing branching (two lines exiting or entering a notebook) or looping as blue and orange arrows (see C). (B) Analysis runs are saved as a stack of directories. AnnData objects may be used in a notebook of a subsequent run, indicated by the arrows. (C) Scheme of an analysis run. Notebooks are run in order (top to bottom) with the option to rerun a notebook (blue arrow; loop) or restart from a prior notebook (orange arrow; loop). The dashed arrows represent potential branches to another run. Running the notebooks automatically populates the folder structure. (D) A grid of embeddings (PCA and UMAP) and their components colored for different metrics over different batch correction tools (columns) and an LISI score plot (bottom row) showing the effect of each tool. (E) UMAP of the 150 dpf sample colored by clusters. See also Figure S2. Subfigures (A–C) created with BioRender.

Unlike most existing workflows (Table S1), the SC-Framework implements self-documenting data objects that automatically record a protocol of all applied analysis steps within the data object itself (see methods for details). The protocol captures function names, timestamps, and corresponding parameters (e.g., filter thresholds, input files), making the full analysis history queryable directly from the data object (Figure 1F). This feature integrates into the AnnData format,^38^ ensuring consistent and traceable data transfer between notebooks and interoperability with external tools. Output files such as figures, tables, and logs are organized through configuration-defined directory structures. Together, these features support iterative analysis: parameters can be revisited and adjusted without losing prior results, and workflows can be branched to compare analytical choices (e.g., batch correction methods) or to diverge from a specific analysis point to bypass computationally intensive steps (Figures 2A–2C). To simplify reproducibility and interoperability, we apply continuous testing routines during development and version the entire code base including notebooks, decoupling execution from environment-specific issues.

The SC-Framework requires computational resources that scale with dataset size. We performed a quantitative benchmark across major notebooks using datasets ranging from fewer than 5,000 cells (typical of 10× Genomics platforms) to nearly 10^6^ cells, measuring RAM usage, disk space, and runtime per notebook (Figure S3 and Table S3), utilizing the containers within a PROXMOX virtualization cluster. All resource requirements increase with the number of cells. Small datasets request hardware needs typically available on local machines such as desktop or laptop computers. Total runtime remains below 10 h even for the largest datasets. Notably, RAM usage varies substantially across analytical steps, with batch correction methods exhibiting particularly high memory demands, reaching up to an order of magnitude greater than those of other computational components (see Table S3 for details).

In summary, the SC-Framework is an FAIR software package that generates reproducible, documented, and FAIR-compliant analytical outputs. It supports entry at intermediate workflow steps, runs locally or on cloud-based infrastructure, and scales from small to large datasets.

### Flexible building blocks enable looping and branching within an scRNA-seq analysis

The SC-Framework allows for flexible analysis runs, including looping and branching, with FAIR data handling.

Looping and branching are integral parts of most analyses, though their ubiquity can make them inconspicuous, as they naturally occur throughout the analysis. In this context, we define looping as re-running parts of the analysis with adjusted parameters, which in traditional workflows requires repeating the entire analysis. The SC-Framework supports this by allowing users to loop back to previous steps at any time, either within a notebook by re-executing code blocks, restarting the current notebook, or jumping back to a previous notebook (Figure 2A). Looping is intended for quickly testing different configurations, with only the most recent configuration retained. In contrast, branching, akin to *git branches*, enables permanent tracking of multiple configurations by splitting the analysis into parallel runs, such as exploring data subsets after QC or combining multiple datasets for joint analysis. Branches are implemented as independent directories, with the first notebook in each run (the assembly notebook) linking to data from the preceding run (Figure 2B). Each notebook accesses a hierarchically organized configuration file (Figure 2C), containing global parameters, default values, relative paths, and resources. Branching can be initiated after any notebook (Figure 2C).

#### Core analysis

The core analysis provides the necessary steps required to prepare a dataset for interpretation. Starting from a raw count matrix (e.g., gene transcript counts per cell for scRNA), it is encapsulated in four modules (Jupyter notebooks; Figure 1C). The four core modules are (1) the assembly, (2) QC, (3) batch correction, and (4) embedding/clustering.

The (1) assembly notebook is intended to reformat the data into the AnnData format^38^ to allow further processing. It is set up to import and handle common SC-related formats from the R ecosystem (Seurat^18^ and SummarizedExperiment^42^), text-based files, typically consisting of three tables (expression-matrix, barcodes, and features, such as often provided by GEO^43^ and other public data repositories), and the Python-ecosystem-related AnnData format.^38^ Multiple files originating from samples, conditions, or multiple datasets are merged into one large object, allowing for flexible integration of arbitrary data points. We added the option to omit or rename variables, observations, and secondary matrix layers to account for inconsistencies created during the merging process, such as outdated QC metrics.

The (2) QC notebook removes low-quality cells and genes (accessibility-related features in case of ATAC-seq) based on common metrics (e.g., total counts and mitochondrial content) (Figure S2A), proposing either a global or a sample-specific threshold using the median absolute deviation or a related method using a Gaussian mixture model (see methods). ATAC-seq-specific parameters can be used as quality proxies, e.g., the fragment length distribution (FLD),^44^ the amount of fragments overlapping with regions of interest, e.g., promoters, the fraction of reads in peaks (FRiP), and the transcription start site enrichment (TSSE). Scores based on external information, such as fragment locations, are optional and omitted or generated according to data availability. Thresholds are customizable by interactive plots. Other commonly used methods, including doublet removal,^13^^,^^14^^,^^45^^,^^46^ ambient RNA denoising^46^^,^^47^ (RNA specific), and the identification and filtering of mitochondrial, ribosomal, or gender genes (mitochondrial and gender chromosomes in ATAC), are optionally available.

In the case of ATAC, peaks are annotated to nearby genes by distance,^48^ which enables gene-related downstream analysis such as gene set enrichment or cell-type annotation. Also, an ATAC-specific feature is the optional binarization of the feature matrix. The chromatin state is often described in a binary notion, as either “open” or “closed.” This has the benefit of negating potential sequencing-depth-related issues.^49^ However, this choice is under debate, as recent studies show that binarization can result in a loss of information as it disregards chromatin differences between alleles.^5^^,^^49^^,^^50^

The (3) batch correction notebook is used to adjust the dataset to focus on factors under investigation by providing methods for normalization and exclusion of unwanted variances (e.g., technical variances). Typically, normalization corrects for sequencing depth and outliers using total counts. The ATAC analog, term frequency-inverse document frequency (TF-IDF), highlights important features based on their occurrence in the dataset. However, recent studies showed that this method should not be treated as a depth-normalization technique but rather as a weighting scheme.^49^ As such, it is suitable for clustering but may not apply to other analysis steps.^49^ The increased sparsity of scATAC influences differences in sequencing depth, which is exacerbated by TF-IDF,^49^ resulting in the correlation of dimension reduction components to total counts,^51^ which we corrected through the component subset (see below).

Subsequently, the optimal set of highly variable genes (HVG) is detected by comparing their mean expression and variance-to-mean ratio to reach the optimal range of 1,000 to 5,000 HVGs.^6^ Our ATAC analog, highly variable features (HVFs), measures the absence of features to accommodate the increased sparsity of scATAC. To further reduce the dimension of the SC expression matrix, we embed it into a low-dimensional space by computing principal-component analysis (PCA; RNA) or latent semantic indexing (LSI; ATAC) on the HVGs^52^/HVFs. To identify components that primarily reflect cellular expression (RNA) or accessibility (ATAC) profiles, we correlate all components with the earlier calculated QC metrics, such as total counts (typically the first one),^51^ mitochondrial content, or cell-cycle phase. We then provide a tool that visually supports the subset of components that are biologically most relevant while collecting enough components to explain sufficient variability (Figures S2B and S2C). Of note, it recommends a subset of components for subsequent filtering and analysis steps. The final component selection is then employed to construct a neighbor graph.

The third notebook ends with a comparative batch correction module. Other studies benchmark a selection of datasets to provide a general guideline on the individual batch tool performance.^53^ However, comparison of multiple batch correction methods in the context of a specific dataset would often be preferable. As such, workflows^54^^,^^55^ were developed to ensure selection of the optimal batch correction tool for an individual dataset by assessing correction performance. To provide this functionality as a fully integrated module, our implementation includes six widely used batch correction tools (BBKNN,^56^ MNN,^57^ Harmony,^9^ Scanorama,^58^ ComBat,^10^ and scVI^24^), as well as plots that illustrate the uncorrected dataset alongside the batch corrected versions (Figure 2D). In addition, we included the Local Inverse Simpson’s Index^9^ (LISI), which measures the “mixedness” of the batches in the embeddings to help select the optimal correction method.

The final (4) clustering notebook features detailed options on computing and fine-tuning embedding and clustering (Figure 2E). It allows for computing UMAP or t-SNE embeddings. To optimize the overall layout, the chosen embedding is computed in a range of parameter configurations from which the optimal representation can be chosen (Figure S2D). Finally, similar to the embedding module, multiple clusterings are calculated and illustrated within the chosen embedding (Figure S2E). Modules enable optional refinement, including cluster splitting or merging (Figure S2F). The final clusters are visualized for selected data layers to evaluate the clustering in terms of biological variance and delimit against technically induced pattern formation (Figures S2G and S2H).

#### Downstream analysis

Based on cell identities and their distribution between different conditions, a plethora of tools are available to perform downstream analyses (Table S2). Several integrated downstream analysis notebooks (Figure 1D) are provided to answer typical biological questions that arise in the context of an SC dataset, which, in contrast to the core notebooks, are not intended to run in a specific order. Most downstream notebooks apply to either RNA- or ATAC-derived data, with exceptions like the velocity analysis, which is exclusive to RNA.

In this regard, two frequently used notebooks are the marker notebook to identify markers, e.g., genes, primarily found in one group, and the annotation notebook to assign groups of cells to categories such as cell types.

The marker identification notebook supports either the Scanpy^19^-provided functionality based on statistical tests (e.g., Wilcoxon) of a dedicated cluster vs. all remaining cells or a module encapsulating DESeq2^59^ for pairwise cluster or condition comparisons. Identified markers are valuable for gene set enrichment analysis (GSEA) or annotation.

The cell-type annotation notebook represents an important step of SC analysis and typically involves computational approaches, such as reference-based (e.g., SingleR^60^ or scmap^61^), marker-based (e.g., CellAssign^62^ or SCSA^17^), or machine-learning-based methods (e.g., SingleCellNet^63^ or TOSICA^64^). These are typically complemented by expert knowledge and manual curation. The SC-Framework supports annotation via SCSA and MarkerRepo (https://github.com/loosolab/MarkerRepo, sub module of SC-Framework). The MarkerRepo implements a reference-based approach, with an interface that facilitates importing, storing, and managing marker lists with optional metadata (e.g., tissue, age, and organism) from databases or publications. Two scoring methods are available to weight marker genes/peaks during annotation. The first is based on the frequency over all selected groups (e.g., cell types), and the second is the ubiquitousness index (UI), as implemented in the PanglaoDB.^65^ Of note, PanglaoDB and other reference databases are restricted mostly to human and mouse genes. Therefore, the MarkerRepo implements additional functionality to automatically transfer marker lists to other species of interest based on a homology database (BioMart^66^ or HomoloGene^67^), enabling the annotation of any organism. Further details are given in the methods section. The annotation notebook is geared to streamline the process of searching and combining marker lists, either supplied by the user or already included within its database.

The multiomics notebook integrates two modalities, such as RNA and ATAC, after each has been individually analyzed through the core analysis notebooks (requiring embedding and clustering). Modalities are linked using Muon^21^ within a MuData object, enabling comparison of modality-specific cell clusters and their alignment across embeddings. An integrated embedding is then computed using MOFA+,^68^ which identifies shared latent factors across modalities, and cells are clustered on this embedding to reveal populations reflecting structure from both modalities. The resulting multimodal clustering is transferred back to the individual AnnData objects and exported alongside the MuData object, preserving compatibility with downstream unimodal analyses while incorporating multimodal insights (see the guidance page and multiomics tutorial in our wiki).

The downstream notebooks also include functionality for proportion analysis, receptor-ligand analysis, gene set enrichment methods, and trajectory and timeline analysis, as well as export of an h5ad object for usage in the CELLxGENE app,^69^ alongside an automated summary report notebook.

### Exemplary scRNA analysis on zebrafish development retraces published results

In this section, we demonstrate the SC-Framework’s ability to retrace the published results of an independent scRNA dataset by Fabian et al.,^70^ which investigates Zebrafish cranial neural crest development across seven time points from embryonic development to adulthood^70^ (Figure 2A). We performed the analysis independently for each time point, combining replicates where applicable (Table S4), and subsequently merged the individual analysis runs to illustrate the framework’s flexibility, including its branching and looping features. The complete analysis was finished within a few hours and is available in detail in our Supplementary Repository (see data availability section).

First, we chose the 150 days post-fertilization (dpf) time point from the zebrafish cranial neural crest dataset and applied our entire core notebook stack. In line with Fabian et al., we retained 6,035 cells after QC (6,099; Table S4). Afterward, the core stack generates a UMAP embedding and initial clustering, assigning cells to putative functional groups (Figure 2E), which also affirm the 22 clusters of Fabian et al., by initial 21 clusters (Table S4).

**Figure 3:**
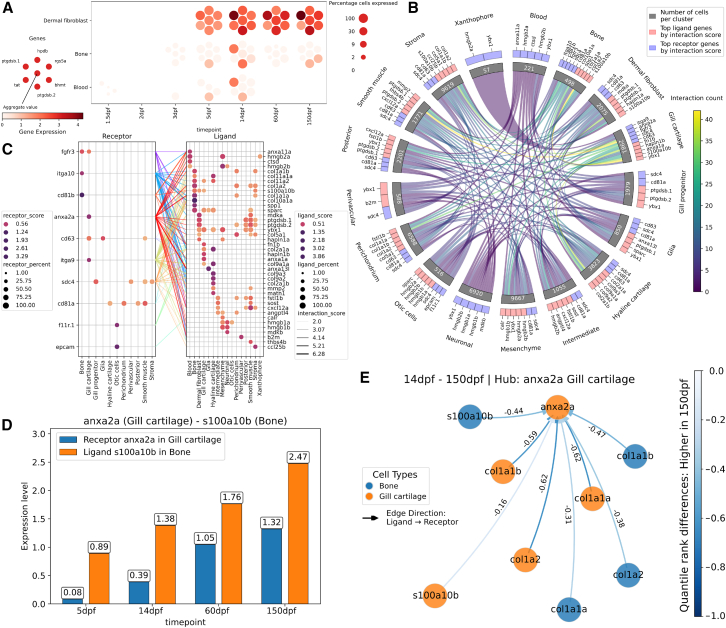
Exemplary scRNA downstream analysis of the combined time points on zebrafish (A) A planet plot showing the gene markers (planets/dots) for a subset of cell types (rows) across time points (columns). The central dot per planet system indicates the mean expression of the moons. (B) Cord plot quantitative interactions between zebrafish cell types. The outermost shell shows the top receptor and ligand genes per cell type. (C) Focused interaction plot presenting a qualitative view of the most prominent receptors (left) connected to their respective ligands (right) between cell types. (D) Single interaction plot for one receptor-ligand pair between specific cell types over time. (E) Hub plot illustrating quantile rank differences of receptor-ligand interactions between 14 and 150 dpf, with genes and clusters selected from (C). Each node represents a gene (receptor or ligand), with interactions and their difference between time points shown as a colored arrow. See also Figure S4.

However, we found these clusters to be suboptimal by investigating the marker panel (Figures S2E, S4A, and S4C) generated by our marker notebook. Looping back to the clustering notebook, we split and recombined into 19 clusters (Figures 2E and S4B), which improved the visual separation of clusters within the UMAP (see the supplementary repository in data availability for details) and subsequent repeated marker detection (Figures S4C and S4D). By assessing individual genes, we confirmed that our notebooks recover well-established cell-type-specific markers. For example, the gene ZFIN: *ucmaa*, described to be primarily expressed in cartilage,^71^ the fibroblast markers ZFIN: *hpdb* and ZFIN: *hgd*,^70^^,^^72^ and the muscle-related genes ZFIN: *thbs4b*^73^ and ZFIN: *myoc*^74^ are all found within the top 15 markers of their respective clusters. Additional *dermal fibroblast* markers defined by Fabian et al. were also found to be expressed in the expected cell type (Figure 3A).

Next, we collected the corresponding markers reported by Fabian et al. (non-mesenchymal CNCC-derived, non-CNCC, 150 dpf) and submitted them to the MarkerRepo for annotation. The annotation notebook optionally runs all algorithms and scoring schemes in parallel, including comparison to an optional reference. In the exemplary 150 dpf dataset, we used the annotation provided by Fabian et al.^70^ as a reference (Figure S4B and Table S4).

Finally, the annotated dataset is stored within the SC-Framework folder structure (Figures 2B and 2C) to become accessible to other notebooks. Here, we looped back to the marker notebook, since the cell-type annotation combined clusters by assigning them to the same cell type (Figure S4B). We then applied the GSEA notebook to find and visualize gene ontology (GO) terms enriched within marker gene sets. As expected, we found GO terms matching the annotated cell types, as reported by Fabian et al. (Figures S4E and S4F).

Next, we performed individual analyses of all other time points similar to the 150 dpf workflow (Figure 2A). In order to demonstrate branching and joining of workflows (Figures 2A–2C), we combined the resulting data objects with the assembly notebook to identify developmental trends during the maturation of zebrafish. Subsequently, we calculated an embedding and clustering via the normalization and clustering notebook for the combined dataset, leaving out QC and batch correction attempts.

Clusters were refined by observing markers, followed by annotation with the combined marker lists of Fabian et al.^70^ The annotated embedding of combined time points was used to investigate developmental changes along the time axis, particularly the emergence of time-point-specific cell types. To answer this question, we utilized the proportion analysis^75^ notebook to calculate significant changes for each cell type per time point (Figure S4G). In agreement with Fabian et al., we found cell types emerging at specific time points, such as *dermal fibroblasts* and *bone* in larvae and later stages.

Finally, we applied our receptor-ligand (RL) analysis to the dataset (see methods), which is intended to predict cell communication between cell types. The notebook provides visualization emphasizing global aspects (e.g., identification of highly interactive cell types supported by a large number of expressed RL pairs; Figure 3B), as well as individual RL interactions showing high activity across >2 cell types (Figure 3C).

For instance, we found the cell-type *gill cartilage* to be highly interactive with most cell types in the dataset (Figure 3B). Upon closer inspection, we found ZFIN: *anxa2a* to be a key gene for these interactions, well known to be involved in processes such as regeneration,^76^^,^^77^^,^^78^ development,^79^^,^^80^ and immune responses,^81^ targeted by various ligands from different cell types. We defined such genes as hub genes and examined potential changes in cellular communication along the timeline. By utilizing the hub notebook, we investigated differences in interactions and found the ZFIN: *anxa2a* hub in *gill cartilage* to follow the expression pattern of its ligand ZFIN: *s100a10b* in *bone* (Figures 3D and 3E). Human orthologs of ZFIN: *anxa2a* and ZFIN: *s100a10b* (NCBI: *AnxA2* and NCBI: *S100A10*) were described to form a complex involved in important biological processes such as exocytosis and membrane repair.^82^ Both genes were suggested to have a function in matrix-vesicle-mediated mineralization, a process needed for bone formation.^83^

In summary, the iterative steps described above underscore how a versatile and resilient workflow can facilitate looping or branching, ensuring the availability of prerequisite data layers, needed for subsequent analysis steps by integration of publicly available and internally implemented tools.

### Generic implementation enables the analysis of different modalities

The SC-Framework is designed and implemented to allow the reuse of large code blocks, modules, and wrappers. It supports applications to a wide range of datasets and data types, as well as quick adaptation to novel developments, i.e., data types. However, some data types, such as scATAC-seq, need data-specific operations.

In order to demonstrate specific steps in this workflow and the types of analysis that are integrated into the framework, we use an exemplary single-nucleus ATAC (snATAC) dataset of human peripheral blood mononuclear cells (PBMCs) from a healthy donor, obtained from 10× Genomics.^84^ The basic steps of the ATAC workflow resemble RNA analysis. The first four notebooks are the core steps, including (1) reformat and assembly, (2) QC steps, (3) batch correction, and (4) embedding and clustering.

We skipped batch correction, as the experimental design did not allow us to discriminate between biological variance, as the dataset contains only one sample/condition (healthy). Consequently, we continued with embedding and clustering (Figure S5A). This concludes the core analysis.

For downstream analyses, we started with the identification of cell types. Genes of the previously annotated peaks were used to identify group markers utilizing the marker notebook as described above for the RNA dataset. The resulting marker genes for each cell cluster were used to annotate cell types with the annotation notebook (Figure S5B). As expected and described by Kleiveland,^85^ we found the PBMC cell types. The amount of each of the cell types matched the expected composition described in the MACS handbook,^86^ except for the *dendritic cells*, which show a higher than expected content (Figure S5C). The increase might be explained by the annotation combining *dendritic cells* and *monocytes* due to their shared lineage.^87^

Next, a differential transcription factor binding analysis was realized using our scTOBIAS^88^ implementation. Cell signals from user-defined groups (e.g., cell type + specific condition) were exported to pseudo bulk cell groups and compared, considering transcription factor binding occupancy. Analysis of the PBMC cell types between *naive CD8*^*+*^ *T cells* and *regulatory T (Treg) cell*s, a derivative of *CD4*^*+*^ *T cells*, identified transcription factors in line with expectations. For example, BACH2,^89^ BATF,^90^ JUNB,^89^^,^^90^ BATF3,^91^ SATB1,^92^^,^^93^ and BACH1^94^ were associated with *CD4*^*+*^ *T cells* and EGR1,^95^ EGR2,^96^ SP1,^97^ and Nrf1^97^ were associated with *CD8*^*+*^ *T cells* (Figure S5D). While most downstream notebooks are data-agnostic, the TOBIAS^88^ notebook is unique to ATAC and not applicable to RNA-seq.

In summary, our ATAC notebooks demonstrate the reuse of shared code across data types, projects of different sizes, and cell numbers.

## Discussion

SC analysis remains complex, relying on field-specific heuristics and the experience of the analyst. Achieving optimal results requires both biological knowledge and computational data exploration, limiting accessibility for users without expertise in either area. Small deviations in workflow or parameter choices can profoundly affect outcomes, posing a persistent challenge to reproducibility.

While several workflow managers and pipeline frameworks exist for SC analysis, ranging from Nextflow-based pipelines such as nf-core/scrnaseq to GUI-driven platforms, these typically prioritize automation and standardization at the expense of interactive exploration and guided interpretation. Conversely, toolkit-based approaches such as Seurat or Scanpy offer flexibility but place the full analytical burden on the user, with no structured guidance or reproducibility scaffolding. The SC-Framework combines both, a standardized reproducible environment with embedded interpretation guidance (notebooks) and the full flexibility of a Python toolkit. Table S1 shows that no listed tool covers all evaluated criteria; the SC Framework was designed to address this gap. It enables the analysis by minimal coding via notebook adaptation, flexible creation of new steps on demand, automatic logging, and structured data storage. Comprehensive documentation, tutorials, and videos support analysts across varying levels of technical expertise, and a Continuous Integration (CI) pipeline including the generation of Docker images ensures robustness and long-term reproducibility.

The framework (and earlier versions of it) has been applied across a broad range of data types and biological contexts in peer-reviewed publications, including scRNA-seq, scATAC-seq, CITE-seq, spatial transcriptomics, immune panel sequencing, and multimodal datasets.^98^^,^^99^^,^^100^^,^^101^^,^^102^^,^^103^^,^^104^^,^^105^^,^^106^^,^^107^^,^^108^^,^^109^^,^^110^^,^^111^^,^^112^^,^^113^^,^^114^ Applications by independent research groups across these modalities indicate that the framework is usable in varied research contexts.

The SC-Framework is actively maintained and deployed within institutional infrastructure. As one example, Docker images are used as standardized analysis environments within the excellence cluster structure of the CPI (https://cpi-online.de/), accessible via a cloud-based frontend for individual use, demonstrating that the containerized fourth layer is deployable beyond local installations. Planned extensions include integration of emerging modalities and analytical methods as they become standardized in the field, as well as continued development of guidance content to keep pace with evolving best practices.

Reproducibility in SC genomics also depends on the analyst. Parameter choices, particularly in steps such as batch correction, clustering resolution, and cell-type annotation, are often not reported in sufficient detail to allow independent reproduction, and the consequences of suboptimal choices are often not apparent until downstream analysis. The SC-Framework embeds interpretation guidance, default parameters, and explicit decision points into the analytical environment, with the aim of making the reasoning behind such choices more transparent.

The SC-Framework processes datasets of nearly 10^6^ cells within the resource limits reported in Table S3, using hardware available at typical research institutes. Larger datasets can be handled through containerized deployment on compute clusters.

### Limitations of the study

Several limitations should be considered. Scalability remains constrained by the computational demands of the underlying tools, particularly for very large datasets or complex multimodal analyses. While the standardized environment reduces dependency management burden, integration of newly emerging methods may lag, as incorporation requires implementation and validation within the framework. Intermediate results are stored in AnnData-based formats to minimize compatibility issues, though export to non-Python or non-AnnData environments may require additional conversion steps.

An important consideration of the SC-Framework is that its self-documentation mechanism captures only the final analytical workflow and not intermediate exploratory steps (e.g., alternative parameter settings), which may limit documentation of the parameter-tuning process.

Finally, parameter choices at all levels of the workflow, particularly in steps such as batch correction and multi-modal integration, can substantially influence downstream results and biological interpretations. The SC-Framework supports both gene-level correction methods (e.g., ComBat and MNN), which adjust expression values directly and preserve interpretability for differential expression analysis, and latent-space methods (e.g., Harmony and BBKNN), which minimize batch effects in low-dimensional representations without modifying the underlying data matrix. Guidance within the notebooks helps users select appropriate strategies for their specific analysis context.

In summary, the SC-Framework provides a centrally maintained, FAIR-compliant analysis environment that balances reproducibility with the flexibility needed to accommodate the evolving demands of the SC field.

## Resource availability

### Lead contact

Requests for further information and resources should be directed to and will be fulfilled by the lead contact, Mario Looso (mario.looso@mpi-bn.mpg.de).

### Materials availability

This study did not generate new unique reagents.

### Data and code availability

This paper analyzes existing, publicly available data. Quantified zebrafish cranial scRNA data of Fabian et al.^70^ were downloaded from GEO accession GSE178969. Human 10k healthy PBMCs used for snATAC analysis are downloaded from 10× Genomics (https://www.10xgenomics.com/datasets/10k-human-pbmcs-atac-v2-chromium-controller-2-standard). See our supplementary GitHub repository for detailed information on the conducted analysis (https://github.com/loosolab/Code_Schultheis_et_al_2025_SC-Framework). All original code is available at GitHub (https://github.com/loosolab/SC-Framework), and each version has been deposited at Zenodo at https://doi.org/10.5281/zenodo.11065517.

## Acknowledgments

This work was supported by the 10.13039/501100001659DFG (Deutsche Forschungsgemeinschaft) under grant ExStra EXC2026 (Translational Hub 2, KFO309 Z Project) to M.L.; 10.13039/501100003495Hessisches Ministerium für Wissenschaft und Kunst (LOEWE iCANx Z Project) to M.L.; DZHK Rhein Main Site; and the 10.13039/501100004189Max Planck Society.

## Author contributions

M.L. conceived the study. M.L., H.S., J.D., R.W., and M.B. designed the software. H.S., J.D., R.W., M.B., Y.A., G.V., M.F.K., B.B., D.M., A.U., K.M., J.W., P.G., M.H., and C.K. implemented the software. H.S. preprocessed and analyzed the data. H.S., J.D., R.W., C.K., and M.L. wrote the manuscript. M.L. supervised the project.

## Declaration of interests

The authors declare no competing interests.

## STAR★Methods

### Key resources table

**Table:** 

REAGENT or RESOURCE	SOURCE	IDENTIFIER
**Software and algorithms**		

Python Version 3.12.12	Python Software Foundation	https://www.python.org/; RRID: SCR_008394
SC-Framework 0.14.2	This paper	https://github.com/loosolab/SC-Framework
MarkerRepo 0.1.5	This paper	https://github.com/loosolab/MarkerRepo

### Method details

#### SC-framework

The SC-Framework includes analysis starting from quantified data (raw counts), over embedding and clustering, to downstream analysis such as cell type annotation or marker identification. It is split into a Python package, providing functionality, and a collection of Jupyter notebooks, detailing the analysis. The SC-Framework with all its resources (documentation, API, etc.) can be accessed on our GitHub (https://github.com/loosolab/SC-Framework).

#### The python package - Sctoolbox

The sctoolbox is a Python package providing the bulk of SC-Framework functionality. It can be installed either through GitHub or the Python Package Index (PyPI). We ensure code quality standards by style and type checks, unit testing, semantic versioning, and change logs. The package provides a full API documentation, including example snippets and respective outputs, and references where applicable.

The sctoolbox features two different logging systems. The traditional logging of custom status messages in different severity levels (info, warning, error, etc.) can be written to a file, directly displayed in the console, or both. The second system is function-level logging, which automatically stores function calls and their parameters in the AnnData.uns attribute of the AnnData object, rendering the object self-documenting and all analysis steps traceable. This is realized using Python Decorators for inclusion of new functions (*sctoolbox.utils.decorator.log_anndata*). This means in a standard use case, that a notebook begins by loading a previously saved.h5ad object, followed by sequential analysis steps whose parameters and results are recorded in AnnData.uns until the AnnData is stored as a.h5ad file and the session is closed. If a user revisits and reruns a previous step within the same notebook (e.g., modifying parameters for a computation such as a UMAP representation), a new session will start and the corresponding entries in AnnData.uns, as well as the derived results, are updated and overwritten to reflect the current state of the analysis. This ensures consistency between stored parameters and the stored data layers in the object. At the end of each notebook, the full set of parameters corresponding to the final state of the data is stored and passed on to subsequent analysis steps via the saved.h5ad object. In this way, each notebook captures a consistent and self-contained snapshot of the analysis state.

The package provides automatic threshold (*sctoolbox.tools.qc_filter.automatic_thresholds*) calculations on cells (AnnData.obs) or features (AnnData.var). Threshold calculation can either be done using the commonly used median absolute deviation (MAD) with independent upper and lower bounds *MAD*_*score*_ = *median*(*v*)±*MAD*×*n*_1, 2_, or a similar function that uses the standard deviation of the largest Gaussian mixture model component *c* = *largestcomponent*(*v*), *GMM*_*score*_ = *mean*(*c*)±*σ*_*c*_×*n*_1, 2_. The function also accepts external threshold functions.

The package comes with a command line based download interface to simplify notebook downloads and analysis set up. The interface further supports version based downloads to facilitate retracing of past analysis.

#### MarkerRepo

The MarkerRepo is a reference annotation tool, implemented within the sctoolbox, to manage genes and other types of markers. It can be accessed using our GitHub (https://github.com/loosolab/MarkerRepo). Its three main topics address common hurdles in the annotation space namely, a searchable database, homology based gene transfer between organisms, and SC oriented annotation algorithms.

The MarkerRepos database is prefilled with several lists spanning multiple organisms of common databases (e.g., PanglaoDB, CATlas, CellMarker) stored in individual lists in YAML format. The format was chosen to store additional metadata in a lightweight, accessible and extendable manner. The metadata contains information such as submission time, source, name and bio-related tags, for example, organism, tissue, disease, life stage. Querying these allows to find lists relevant to the dataset and to combine them for annotation in a resolution suitable for the current investigation. The database can contain lists of arbitrary groups such as cell types, for transcriptomics, or regulatory elements, typically investigated in chromatin context.

Organisms with no available marker databases can be annotated through homology-based gene transfer. The MarkerRepo implements access to query the BioMart API or to HomoloGene utilizing a local database as an offline alternative.

Two different annotation algorithms are available within the MarkerRepo. A reimplementation of SCSA that assign groups of cells to cell types, by computing a score based on differential gene expression and the marker evidence of each gene. The MarkerRepo internal algorithm annotates by scoring aa cell types fit to a group through summing the rank scores, computed by Scanpy’s ranked_genes_groups function, of genes matching the marker database. The rank scores are further multiplied by the ubiquitousness index (UI), of PanglaoDB, if they are available, to reduce the influence of genes appearing in multiple different cell types of the database. This is divided by the square root of total marker genes of the respective cell type in the database to reduce the influence of cell types with a high number of genes. The result is a score per cell type available in the database for each group of cells. The scores are then scaled using the adjacent disparity to identify whether any of the possible cell types fits significantly better than the rest to the cell group, causing it to be assigned to this group.

#### Receptor-ligand analysis

The RL analysis requires a database with receptor and ligand pairings to proceed. We integrated the LIANA+^115^ package to provide multiple databases of different organisms. A custom database, in the form of a table with a column of receptor-gene names and a column of corresponding ligand-gene names, may be used as an alternative. Interactions are scored for their combined receptor and ligand enrichment between clusters of cells, adjusted for the cluster size, and scaled for the number of cells expressing the respective gene. The interaction score of a specific interaction is calculated as the sum of the participating receptor and ligand gene scores. The gene scores are calculated as the *Z* score over the cluster mean expressions, multiplied by the proportion of cells assigned to the respective cluster, and multiplied by the proportion of cells within the cluster expressing the gene. The interaction score is based on the *w*_2_-score of Brickman et al.^116^ Interaction scores >0 can be interpreted as “enriched”, while <0 means “depleted”.

The identification of changing interactions between conditions is investigated through the quantile-ranked differences. This is realized by calculating the interaction scores for each condition by combining and ranking the interactions. The difference in rank between the two conditions yields the final score for every interaction. Conditions are implemented to be chosen arbitrarily and can be nested. For example, the data may be separated into wild type and multiple treatments, and on a second level, subdivided into patients. It is also possible to define an order for conditions with an inherent sequence, such as timepoints, to restrict the difference comparisons to the previous and directly following condition.

#### Benchmark

A quantitative benchmark is conducted on datasets spanning 3020 to 836569 cells. The benchmark was carried out on a common workflow applicable to most datasets, starting with the core analysis (assembly, QC, batch correction, clustering notebooks) and proceeding with group marker and cell type annotation. The notebooks were executed in the mentioned order while tracking memory, disk space usage and runtime (Figure S3 and Table S3). The tests were run in PROXMOX virtualization environment (Version 8.4) with six nodes, each with 72 cores (base clock speed 2.2 GHz) and two terabytes of memory, using our docker containers (https://gitlab.gwdg.de/loosolab/software/sc_framework/container_registry/).

### Quantification and statistical analysis

Pre-analyzed datasets were obtained from public ressources, namely count matrices. The analysis workflows were conducted with SC-Framework version 0.14.2. Integrated software within SC-Framework can be found in Table S2. The detailed analysis steps and individual software versions can be accessed in the supplementary code repository (https://github.com/loosolab/Code_Schultheis_et_al_2025_SC-Framework).

## References

[1] Ning L., Liu G., Li G., Hou Y., Tong Y., He J. (2014). Current Challenges in the Bioinformatics of Single Cell Genomics. Front. Oncol..

[2] Zappia L., Theis F.J. (2021). Over 1000 tools reveal trends in the single-cell RNA-seq analysis landscape. Genome Biol..

[3] Amezquita R.A., Lun A.T.L., Becht E., Carey V.J., Carpp L.N., Geistlinger L., Marini F., Rue-Albrecht K., Risso D., Soneson C. (2020). Orchestrating single-cell analysis with Bioconductor. Nat. Methods.

[4] Grones C., Eekhout T., Shi D., Neumann M., Berg L.S., Ke Y., Shahan R., Cox K.L., Gomez-Cano F., Nelissen H. (2024). Best practices for the execution, analysis, and data storage of plant single-cell/nucleus transcriptomics. Plant Cell.

[5] Heumos L., Schaar A.C., Lance C., Litinetskaya A., Drost F., Zappia L., Lücken M.D., Strobl D.C., Henao J., Curion F. (2023). Best practices for single-cell analysis across modalities. Nat. Rev. Genet..

[6] Luecken M.D., Theis F.J. (2019). Current best practices in single-cell RNA-seq analysis: a tutorial. Mol. Syst. Biol..

[7] Kaminow B., Yunusov D., Dobin A. (2021). STARsolo: accurate, fast and versatile mapping/quantification of single-cell and single-nucleus RNA-seq data. bioRxiv.

[8] Zheng G.X.Y., Terry J.M., Belgrader P., Ryvkin P., Bent Z.W., Wilson R., Ziraldo S.B., Wheeler T.D., McDermott G.P., Zhu J. (2017). Massively parallel digital transcriptional profiling of single cells. Nat. Commun..

[9] Korsunsky I., Millard N., Fan J., Slowikowski K., Zhang F., Wei K., Baglaenko Y., Brenner M., Loh P.R., Raychaudhuri S. (2019). Fast, sensitive and accurate integration of single-cell data with Harmony. Nat. Methods.

[10] Leek J.T., Johnson W.E., Parker H.S., Jaffe A.E., Storey J.D. (2012). The sva package for removing batch effects and other unwanted variation in high-throughput experiments. Bioinformatics.

[11] Traag V.A., Waltman L., van Eck N.J. (2019). From Louvain to Leiden: guaranteeing well-connected communities. Sci. Rep..

[12] DeTomaso D., Yosef N. (2021). Hotspot identifies informative gene modules across modalities of single-cell genomics. Cell Syst..

[13] Wolock S.L., Lopez R., Klein A.M. (2019). Scrublet: Computational Identification of Cell Doublets in Single-Cell Transcriptomic Data. Cell Syst..

[14] Thibodeau A., Eroglu A., McGinnis C.S., Lawlor N., Nehar-Belaid D., Kursawe R., Marches R., Conrad D.N., Kuchel G.A., Gartner Z.J. (2021). AMULET: a novel read count-based method for effective multiplet detection from single nucleus ATAC-seq data. Genome Biol..

[15] Faure L., Soldatov R., Kharchenko P.V., Adameyko I. (2023). scFates: a scalable python package for advanced pseudotime and bifurcation analysis from single-cell data. Bioinformatics.

[16] Setty M., Kiseliovas V., Levine J., Gayoso A., Mazutis L., Pe'er D. (2019). Characterization of cell fate probabilities in single-cell data with Palantir. Nat. Biotechnol..

[17] Cao Y., Wang X., Peng G. (2020). SCSA: A Cell Type Annotation Tool for Single-Cell RNA-seq Data. Front. Genet..

[18] Hao Y., Stuart T., Kowalski M.H., Choudhary S., Hoffman P., Hartman A., Srivastava A., Molla G., Madad S., Fernandez-Granda C., Satija R. (2024). Dictionary learning for integrative, multimodal and scalable single-cell analysis. Nat. Biotechnol..

[19] Wolf F.A., Angerer P., Theis F.J. (2018). SCANPY: large-scale single-cell gene expression data analysis. Genome Biol..

[20] Danese A., Richter M.L., Chaichoompu K., Fischer D.S., Theis F.J., Colomé-Tatché M. (2021). EpiScanpy: integrated single-cell epigenomic analysis. Nat. Commun..

[21] Bredikhin D., Kats I., Stegle O. (2022). MUON: multimodal omics analysis framework. Genome Biol..

[22] Palla G., Spitzer H., Klein M., Fischer D., Schaar A.C., Kuemmerle L.B., Rybakov S., Ibarra I.L., Holmberg O., Virshup I. (2022). Squidpy: a scalable framework for spatial omics analysis. Nat. Methods.

[23] Zhang K., Zemke N.R., Armand E.J., Ren B. (2024). A fast, scalable and versatile tool for analysis of single-cell omics data. Nat. Methods.

[24] Gayoso A., Lopez R., Xing G., Boyeau P., Valiollah Pour Amiri V., Hong J., Wu K., Jayasuriya M., Mehlman E., Langevin M. (2022). A Python library for probabilistic analysis of single-cell omics data. Nat. Biotechnol..

[25] Adil A., Kumar V., Jan A.T., Asger M. (2021). Single-Cell Transcriptomics: Current Methods and Challenges in Data Acquisition and Analysis. Front. Neurosci..

[26] Lu J., Sheng Y., Qian W., Pan M., Zhao X., Ge Q. (2023). scRNA-seq data analysis method to improve analysis performance. IET Nanobiotechnol..

[27] Lee J., Christoforo G., Christoforo G., Foo C.S., Probert C., Kundaje A., Boley N., Kohpangwei, Dacre M., Kim D. (2016). kundajelab/atac_dnase_pipelines: 0.3.3. Zenodo.

[28] Hitz B.C., Jin-Wook L., Jolanki O., Kagda M.S., Graham K., Sud P., Gabdank I., Strattan J.S., Sloan C.A., Dreszer T. (2023). The ENCODE Uniform Analysis Pipelines. bioRxiv.

[29] Ewels P.A., Peltzer A., Fillinger S., Patel H., Alneberg J., Wilm A., Garcia M.U., Di Tommaso P., Nahnsen S. (2020). The nf-core framework for community-curated bioinformatics pipelines. Nat. Biotechnol..

[30] Trull A., Worthey E.A., Ianov L. (2025). scnanoseq: an nf-core pipeline for Oxford Nanopore single-cell RNA-sequencing. Bioinformatics.

[31] Almeida F. M. de, Peltzer A., Sturm G., Heylf, Botvinnik O., He D., Trummer N., Menden K., Talbot A., nf-core bot (2025). nf-core/scrnaseq: 4.0.0. Zenodo.

[32] Hoek A., Maibach K., Özmen E., Vazquez-Armendariz A.I., Mengel J.P., Hain T., Herold S., Goesmann A. (2021). WASP: a versatile, web-accessible single cell RNA-Seq processing platform. BMC Genom..

[33] Yu H., Wang Y., Zhang X., Wang Z. (2022). GRACE: a comprehensive web-based platform for integrative single-cell transcriptome analysis. NAR Genom Bioinform.

[34] Gardeux V., David F.P.A., Shajkofci A., Schwalie P.C., Deplancke B. (2017). ASAP: a web-based platform for the analysis and interactive visualization of single-cell RNA-seq data. Bioinformatics.

[35] Tzaferis C., Karatzas E., Baltoumas F.A., Pavlopoulos G.A., Kollias G., Konstantopoulos D. (2023). SCALA: A complete solution for multimodal analysis of single-cell Next Generation Sequencing data. Comput. Struct. Biotechnol. J..

[36] Wilkinson M.D., Dumontier M., Aalbersberg I.J.J., Appleton G., Axton M., Baak A., Blomberg N., Boiten J.W., da Silva Santos L.B., Bourne P.E. (2016). The FAIR Guiding Principles for scientific data management and stewardship. Sci. Data.

[37] Barker M., Chue Hong N.P., Katz D.S., Lamprecht A.L., Martinez-Ortiz C., Psomopoulos F., Harrow J., Castro L.J., Gruenpeter M., Martinez P.A., Honeyman T. (2022). Introducing the FAIR Principles for research software. Sci. Data.

[38] Virshup I., Rybakov S., Theis F.J., Angerer P., Wolf F.A. (2024). anndata: Access and store annotated data matrices. J. Open Source Softw..

[39] Jovic D., Liang X., Zeng H., Lin L., Xu F., Luo Y. (2022). Single-cell RNA sequencing technologies and applications: A brief overview. Clin. Transl. Med..

[40] Lafzi A., Moutinho C., Picelli S., Heyn H. (2018). Tutorial: guidelines for the experimental design of single-cell RNA sequencing studies. Nat. Protoc..

[41] Merkel D. (2014). Docker: lightweight Linux containers for consistent development and deployment. Linux J..

[42] Morgan M., Obenchain V., Hester J., Pagès H. (2025). SummarizedExperiment: A container (S4 class) for matrix-like assays. Bioconductor. http://bioconductor.org/packages/SummarizedExperiment/.

[43] Barrett T., Wilhite S.E., Ledoux P., Evangelista C., Kim I.F., Tomashevsky M., Marshall K.A., Phillippy K.H., Sherman P.M., Holko M. (2013). NCBI GEO: archive for functional genomics data sets—update. Nucleic Acids Res..

[44] Detleffsen J., Bruns B., Bentsen M., Kuenne C., Looso M. (2025). PEAKQC: periodicity evaluation in single-cell ATAC-seq data for quality assessment. Brief. Bioinform..

[45] Xi N.M., Li J.J. (2021). Benchmarking Computational Doublet-Detection Methods for Single-Cell RNA Sequencing Data. Cell Syst..

[46] Gondal M.N., Shah S.U.R., Chinnaiyan A.M., Cieslik M. (2024). A systematic overview of single-cell transcriptomics databases, their use cases, and limitations. Front. Bioinform..

[47] Sheng C., Lopes R., Li G., Schuierer S., Waldt A., Cuttat R., Dimitrieva S., Kauffmann A., Durand E., Galli G.G. (2022). Probabilistic machine learning ensures accurate ambient denoising in droplet-based single-cell omics. bioRxiv.

[48] Kondili M., Fust A., Preussner J., Kuenne C., Braun T., Looso M. (2017). UROPA: a tool for Universal RObust Peak Annotation. Sci. Rep..

[49] Kwok A.W.C., Shim H., McCarthy D.J. (2024). Going beyond cell clustering and feature aggregation: Is there single cell level information in single-cell ATAC-seq data?. bioRxiv.

[50] Martens L.D., Fischer D.S., Yépez V.A., Theis F.J., Gagneur J. (2024). Modeling fragment counts improves single-cell ATAC-seq analysis. Nat. Methods.

[51] Cusanovich D.A., Reddington J.P., Garfield D.A., Daza R.M., Aghamirzaie D., Marco-Ferreres R., Pliner H.A., Christiansen L., Qiu X., Steemers F.J. (2018). The cis-regulatory dynamics of embryonic development at single-cell resolution. Nature.

[52] Heimberg G., Bhatnagar R., El-Samad H., Thomson M. (2016). Low Dimensionality in Gene Expression Data Enables the Accurate Extraction of Transcriptional Programs from Shallow Sequencing. Cell Syst..

[53] Tran H.T.N., Ang K.S., Chevrier M., Zhang X., Lee N.Y.S., Goh M., Chen J. (2020). A benchmark of batch-effect correction methods for single-cell RNA sequencing data. Genome Biol..

[54] Luecken M.D., Büttner M., Chaichoompu K., Danese A., Interlandi M., Mueller M.F., Strobl D.C., Zappia L., Dugas M., Colomé-Tatché M., Theis F.J. (2022). Benchmarking atlas-level data integration in single-cell genomics. Nat. Methods.

[55] Chazarra-Gil R., van Dongen S., Kiselev V.Y., Hemberg M. (2021). Flexible comparison of batch correction methods for single-cell RNA-seq using BatchBench. Nucleic Acids Res..

[56] Polański K., Young M.D., Miao Z., Meyer K.B., Teichmann S.A., Park J.E. (2020). BBKNN: fast batch alignment of single cell transcriptomes. Bioinformatics.

[57] Haghverdi L., Lun A.T.L., Morgan M.D., Marioni J.C. (2018). Batch effects in single-cell RNA-sequencing data are corrected by matching mutual nearest neighbors. Nat. Biotechnol..

[58] Hie B., Bryson B., Berger B. (2019). Efficient integration of heterogeneous single-cell transcriptomes using Scanorama. Nat. Biotechnol..

[59] Love M.I., Huber W., Anders S. (2014). Moderated estimation of fold change and dispersion for RNA-seq data with DESeq2. Genome Biol..

[60] Aran D., Looney A.P., Liu L., Wu E., Fong V., Hsu A., Chak S., Naikawadi R.P., Wolters P.J., Abate A.R. (2019). Reference-based analysis of lung single-cell sequencing reveals a transitional profibrotic macrophage. Nat. Immunol..

[61] Kiselev V.Y., Yiu A., Hemberg M. (2018). scmap: projection of single-cell RNA-seq data across data sets. Nat. Methods.

[62] Zhang A.W., O'Flanagan C., Chavez E.A., Lim J.L.P., Ceglia N., McPherson A., Wiens M., Walters P., Chan T., Hewitson B. (2019). Probabilistic cell-type assignment of single-cell RNA-seq for tumor microenvironment profiling. Nat. Methods.

[63] Tan Y., Cahan P. (2019). SingleCellNet: A Computational Tool to Classify Single Cell RNA-Seq Data Across Platforms and Across Species. Cell Syst..

[64] Chen J., Xu H., Tao W., Chen Z., Zhao Y., Han J.D.J. (2023). Transformer for one stop interpretable cell type annotation. Nat. Commun..

[65] Franzén O., Gan L.-M., Björkegren J.L.M. (2019). PanglaoDB: a web server for exploration of mouse and human single-cell RNA sequencing data. Database..

[66] Smedley D., Haider S., Ballester B., Holland R., London D., Thorisson G., Kasprzyk A. (2009). BioMart – biological queries made easy. BMC Genom..

[67] NCBI Resource Coordinators (2014). Database resources of the National Center for Biotechnology Information. Nucleic Acids Res..

[68] Argelaguet R., Arnol D., Bredikhin D., Deloro Y., Velten B., Marioni J.C., Stegle O. (2020). MOFA+: a statistical framework for comprehensive integration of multi-modal single-cell data. Genome Biol..

[69] Megill C., Martin B., Weaver C., Bell S., Prins L., Badajoz S., McCandless B., Pisco A.O., Kinsella M., Griffin F. (2021). cellxgene: a performant, scalable exploration platform for high dimensional sparse matrices. bioRxiv.

[70] Fabian P., Tseng K.C., Thiruppathy M., Arata C., Chen H.J., Smeeton J., Nelson N., Crump J.G. (2022). Lifelong single-cell profiling of cranial neural crest diversification in zebrafish. Nat. Commun..

[71] Neacsu C.D., Grosch M., Tejada M., Winterpacht A., Paulsson M., Wagener R., Tagariello A. (2011). Ucmaa (Grp-2) is required for zebrafish skeletal development. Evidence for a functional role of its glutamate γ-carboxylation. Matrix Biol..

[72] Rajan A.M., Rosin N.L., Labit E., Biernaskie J., Liao S., Huang P. (2023). Single-cell analysis reveals distinct fibroblast plasticity during tenocyte regeneration in zebrafish. Sci. Adv..

[73] Subramanian A., Schilling T.F. (2014). Thrombospondin-4 controls matrix assembly during development and repair of myotendinous junctions. eLife.

[74] Whitesell T.R., Chrystal P.W., Ryu J.R., Munsie N., Grosse A., French C.R., Workentine M.L., Li R., Zhu L.J., Waskiewicz A. (2019). foxc1 is required for embryonic head vascular smooth muscle differentiation in zebrafish. Dev. Biol..

[75] Alayoubi Y., Bentsen M., Looso M. (2024). Scanpro is a tool for robust proportion analysis of single-cell resolution data. Sci. Rep..

[76] Saxena S., Purushothaman S., Meghah V., Bhatti B., Poruri A., Meena Lakshmi M.G., Sarath Babu N., Narasimha Murthy C.L., Mandal K.K., Kumar A. (2016). Role of annexin gene and its regulation during zebrafish caudal fin regeneration. Wound Repair Regen..

[77] Liu W., Hajjar K.A. (2016). The annexin A2 system and angiogenesis. Biol. Chem..

[78] Shi S., Zhang Q., Zhang K., Chen W., Xie H., Pan S., Xue Z., You B., Zhao J., You Y. (2024). FGF19 promotes nasopharyngeal carcinoma progression by inducing angiogenesis via inhibiting TRIM21-mediated ANXA2 ubiquitination. Cell. Oncol..

[79] Partevian S.A., Safina D.R., Rudenok M.M., Rybolovlev I.N., Semenova E.I., Shadrina M.I., Slominsky P.A., Kostrov S.V., Alieva A.K. (2023). The Effect of Morpholino Oligonucleotides to Gene Anxa2a on the Embryonic Development of Danio rerio. Mol. Genet. Microbiol. Virol..

[80] Zhang M., Li X., Cui X., Li R., Ma Z., Gao X. (2023). Selenomethionine promotes ANXA2 phosphorylation for proliferation and protein synthesis of myoblasts and skeletal muscle growth. J. Nutr. Biochem..

[81] Li J., Wang L., Zhang X., Wen X., Wei X., Qin Q., Wang S. (2023). Grouper annexin A2 affects RGNNV by regulating the host immune response. Fish Shellfish Immunol..

[82] Bharadwaj A., Kempster E., Waisman D.M. (2021). The Annexin A2/S100A10 Complex: The Mutualistic Symbiosis of Two Distinct Proteins. Biomolecules.

[83] Cmoch A., Strzelecka-Kiliszek A., Palczewska M., Groves P., Pikula S. (2011). Matrix vesicles isolated from mineralization-competent Saos-2 cells are selectively enriched with annexins and S100 proteins. Biochem. Biophys. Res. Commun..

[84] 10k Human PBMCs, ATAC v2, Chromium Controller (2022). 10x Genomics. https://www.10xgenomics.com/datasets/10k-human-pbmcs-atac-v2-chromium-controller-2-standard.

[85] Kleiveland C.R. (2015). The Impact of Food Bioactives on Health: in vitro and ex vivo models.

[86] Peripheral Blood | Whole blood | Handbook | Miltenyi Biotec | Deutschland. https://www.miltenyibiotec.com/DE-en/support/macs-handbook/human-cells-and-organs/human-cell-sources/blood-human.html.

[87] Liu K., Nussenzweig M.C. (2010). Origin and development of dendritic cells. Immunol. Rev..

[88] Bentsen M., Goymann P., Schultheis H., Klee K., Petrova A., Wiegandt R., Fust A., Preussner J., Kuenne C., Braun T. (2020). ATAC-seq footprinting unravels kinetics of transcription factor binding during zygotic genome activation. Nat. Commun..

[89] Zwick D., Vo M.T., Shim Y.J., Reijonen H., Do J.S. (2024). BACH2: The Future of Induced T-Regulatory Cell Therapies. Cells.

[90] Titcombe P.J., Silva Morales M., Zhang N., Mueller D.L. (2023). BATF represses BIM to sustain tolerant T cells in the periphery. J. Exp. Med..

[91] Patterson A.M., Nakano H., Whitehead G.S., Wilkinson C.L., Nakano K., Massri A.J., Cook D.N. (2025). Lung-resident memory CD4+ T cells are dependent on Batf3. J. Immunol..

[92] Wang B., Bian Q. (2024). SATB1 prevents immune cell infiltration by regulating chromatin organization and gene expression of a chemokine gene cluster in T cells. Commun. Biol..

[93] Gupta P.K., Allocco J.B., Fraipont J.M., McKeague M.L., Wang P., Andrade M.S., McIntosh C., Chen L., Wang Y., Li Y. (2022). Reduced Satb1 expression predisposes CD4+ T conventional cells to Treg suppression and promotes transplant survival. Proc. Natl. Acad. Sci. USA.

[94] Wei X., He Y., Yu Y., Tang S., Liu R., Guo J., Jiang Q., Zhi X., Wang X., Meng D. (2025). The Multifaceted Roles of BACH1 in Disease: Implications for Biological Functions and Therapeutic Applications. Adv. Sci..

[95] Song J., Lu Y., Liu L., Han X., Meng Y., Heng B.C., Zhang X., Cui Q., Liu Z., Guo Y. (2025). Charged substrate treatment enhances T cell mediated cancer immunotherapy. Nat. Commun..

[96] Wagle M.V., Vervoort S.J., Kelly M.J., Van Der Byl W., Peters T.J., Martin B.P., Martelotto L.G., Nüssing S., Ramsbottom K.M., Torpy J.R. (2021). Antigen-driven EGR2 expression is required for exhausted CD8+ T cell stability and maintenance. Nat. Commun..

[97] Moskowitz D.M., Zhang D.W., Hu B., Le Saux S., Yanes R.E., Ye Z., Buenrostro J.D., Weyand C.M., Greenleaf W.J., Goronzy J.J. (2017). Epigenomics of human CD8 T cell differentiation and aging. Sci. Immunol..

[98] Tombor L.S., John D., Glaser S.F., Luxán G., Forte E., Furtado M., Rosenthal N., Baumgarten N., Schulz M.H., Wittig J. (2021). Single cell sequencing reveals endothelial plasticity with transient mesenchymal activation after myocardial infarction. Nat. Commun..

[99] Khassafi F., Chelladurai P., Valasarajan C., Nayakanti S.R., Martineau S., Sommer N., Yokokawa T., Boucherat O., Kamal A., Kiely D.G. (2023). Transcriptional profiling unveils molecular subgroups of adaptive and maladaptive right ventricular remodeling in pulmonary hypertension. Nat. Cardiovasc. Res..

[100] Dizdarevic S., Wiegandt R., Turkowski K., Seeger W., Weigert A., Pullamsetti S., Looso M., Savai R. (2025). Mapping the Immune Landscape of Lung Cancer: Identification of Unique CD45+ Cell Subsets. Am. J. Respir. Crit. Care Med..

[101] Panza P., Kim H.T., Lautenschläger T., Piesker J., Günther S., Alayoubi Y., Cleaver O., Looso M., Stainier D.Y.R. (2025). The lung microvasculature promotes alveolar type 2 cell differentiation via secreted SPARCL1. Stem Cell Rep..

[102] Xu Y., Gehlot R., Capon S.J., Albu M., Gretz J., Bloomekatz J., Mattonet K., Vucicevic D., Talyan S., Kikhi K. (2024). PDGFRA is a conserved HAND2 effector during early cardiac development. Nat. Cardiovasc. Res..

[103] Wang Z.-Y., Mehra A., Wang Q.C., Gupta S., Ribeiro da Silva A., Juan T., Günther S., Looso M., Detleffsen J., Stainier D.Y.R., Marín-Juez R. (2024). flt1 inactivation promotes zebrafish cardiac regeneration by enhancing endothelial activity and limiting the fibrotic response. Development.

[104] Goumenaki P., Günther S., Kikhi K., Looso M., Marín-Juez R., Stainier D.Y.R. (2024). The innate immune regulator MyD88 dampens fibrosis during zebrafish heart regeneration. Nat. Cardiovasc. Res..

[105] Malainou C., Peteranderl C., Ferrero M.R., Vazquez-Armendariz A.I., Alexopoulos I., Franz K., Knippenberg K., Better J., Estiri M., Wu C. (2026). TNF Superfamily Member 14 Drives Post-Influenza Depletion of Alveolar Macrophages Enabling Secondary Pneumococcal Pneumonia. J. Clin. Invest..

[106] Jang J., Bentsen M., Kim Y.J., Kim E., Garg V., Cai C.L., Looso M., Li D. (2024). Endocardial HDAC3 is required for myocardial trabeculation. Nat. Commun..

[107] da Silva A.R., Gunawan F., Boezio G.L.M., Faure E., Théron A., Avierinos J.F., Lim S., Jha S.G., Ramadass R., Guenther S. (2024). egr3 is a mechanosensitive transcription factor gene required for cardiac valve morphogenesis. Sci. Adv..

[108] Cardeira-da-Silva J., Wang Q., Sagvekar P., Mintcheva J., Latting S., Günther S., Ramadass R., Yekelchyk M., Preussner J., Looso M. (2024). Antigen presentation plays positive roles in the regenerative response to cardiac injury in zebrafish. Nat. Commun..

[109] Juan T., Bellec M., Cardoso B., Athéa H., Fukuda N., Albu M., Günther S., Looso M., Stainier D.Y.R. (2024). Control of cardiac contractions using Cre-lox and degron strategies in zebrafish. Proc. Natl. Acad. Sci..

[110] Roquid K.A., Vucetic A., Dyukova E., Hanssen M.J., Cho H., Bonnavion R., Mattonet K., Looso M., Sanda M., Strilic B., Offermanns S. (2025). Lung endothelial PEAR1 induces tumor cell dormancy. Mol. Cancer.

[111] Chu X., Kheirollahi V., Lingampally A., Chelladurai P., Valasarajan C., Vazquez-Armendariz A.I., Hadzic S., Khadim A., Pak O., Rivetti S. (2024). GLI1+ Cells Contribute to Vascular Remodeling in Pulmonary Hypertension. Circ. Res..

[112] Candlish M., Hofmann J., Brösamle D., Haessler A., DeMeglio M., Skodras A., Tushev G., De Biasi E.S., Günther S., Wiegandt R. (2026). Ischemic injury triggers a protective microglial phenotype in models of Aβ pathology. J. Neuroinflammation.

[113] Gupta S., Bajwa G.K., El-Sammak H., Mattonet K., Günther S., Looso M., Stainier D.Y.R., Marín-Juez R. (2025). The transmembrane glycoprotein Gpnmb is required for the immune and fibrotic responses during zebrafish heart regeneration. Dev. Biol..

[114] Tarasco M., Juan T., Alayoubi Y., Kulkarni R., Schumann A., Ribeiro da Silva A., Albu M., Dwari M., Dlnija M., Günther S. (2026). Time-resolved single-cell transcriptomics maps zebrafish heart development. Cell Rep..

[115] Dimitrov D., Schäfer P.S.L., Farr E., Rodriguez-Mier P., Lobentanzer S., Badia-I-Mompel P., Dugourd A., Tanevski J., Ramirez Flores R.O., Saez-Rodriguez J. (2024). LIANA+ provides an all-in-one framework for cell–cell communication inference. Nat. Cell Biol..

[116] Raredon M.S.B., Yang J., Garritano J., Wang M., Kushnir D., Schupp J.C., Adams T.S., Greaney A.M., Leiby K.L., Kaminski N. (2022). Computation and visualization of cell–cell signaling topologies in single-cell systems data using Connectome. Sci. Rep..

